# Do dogs form reputations of humans? No effect of age after indirect and direct experience in a food-giving situation

**DOI:** 10.1007/s10071-025-01967-w

**Published:** 2025-06-28

**Authors:** Hoi-Lam Jim, Kadisha Belfiore, Eva B. Martinelli, Mayte Martínez, Friederike Range, Sarah Marshall-Pescini

**Affiliations:** 1 Domestication Lab, Konrad Lorenz Institute of Ethology, Department of Interdisciplinary Life Sciences, University of Veterinary Medicine Vienna, Vienna, Austria; 2 Present Address: Institute for the Future of Human Society, Kyoto University, Kyoto, Japan; 3 Japan Society for the Promotion of Science, Tokyo, Japan; 4 Present Address: Department of Psychology, University of Michigan, Ann Arbor, MI USA

**Keywords:** Dog cognition, Social cognition, Eavesdropping, Reputation formation, Age, Human-animal interactions

## Abstract

Animals can form reputations of individuals through direct interactions or by observing interactions with a third party, known as eavesdropping. Given their cooperative relationship with humans, considerable interest has focused on whether dogs (*Canis lupus familiaris*) can socially evaluate humans, though findings remain mixed. To assess whether this ability develops during ontogeny, we investigated whether dogs of different ages (young, adult, and senior) can form reputations of humans after observing them interact with a conspecific or through direct interactions in a food-giving situation. Forty pet dogs participated in the experiment. In the eavesdropping condition, dogs observed two humans interact with a dog demonstrator—one was generous and fed the dog, while the other was selfish and withheld food. In the direct experience condition, dogs interacted with the two partners directly. We analysed dogs’ first choice and time spent exhibiting affiliative behaviours towards each partner. Results showed that dogs across all age groups did not significantly prefer the generous partner compared to the selfish partner, nor did their behaviour exceed chance levels following indirect or direct experience. These findings do not provide support for dogs showing reputation formation and highlight the methodological complexities of studying this phenomenon.

**Supplementary Information:**

The online version contains supplementary material available at 10.1007/s10071-025-01967-w.

## Introduction

Group-living animals often cooperate to increase their chance of survival; for example, wolves (*Canis lupus*) work together to hunt large prey (MacNulty et al. 2014). Cooperation provides mutual benefits to all individuals involved, such as shared access to resources, but challenges arise when individuals are not willing to share the resources obtained through cooperation or when free riders exploit the benefits without contributing. Therefore, animals may benefit from being able to socially evaluate individuals.

Reputation refers to knowledge about an individual’s typical behaviour based on their past actions (Russell et al. 2008). Animals can form reputations either by directly interacting with each other or by observing third-party interactions, known as eavesdropping or third-party evaluation (Subiaul et al. 2008). Although judgements based on direct interactions are more accurate, eavesdropping avoids the potential costs of direct experience, such as physical harm from aggressive individuals. However, eavesdropping is cognitively demanding, as it requires recognising and remembering behaviours observed in third-party interactions, as well as the individuals performing them. Despite this complexity, the ability to eavesdrop is particularly useful for group-living animals, as it may facilitate cooperation by enabling them to choose ‘good’ partners.

Typically, reputation formation in animals is studied using the general procedure of a demonstration phase followed by a test phase. In the demonstration phase, the subject interacts (direct reputation formation) or observes another individual interacting (eavesdropping) with two partners with different roles, such as a generous/helpful vs. a selfish/unhelpful partner. Then, in the test phase, the subject can choose with whom to interact. Usually, the partners in such interactions are humans, since, despite training, it is difficult to guarantee that animals will consistently act in a specific manner over repeated trials (e.g., one partner acting generously and the other selfishly).

Comparative cognition studies have mainly focused on non-human primates, our closest living relatives, to investigate whether complex sociocognitive abilities like eavesdropping are unique to humans. Research on non-human primates’ social evaluation skills have produced mixed results (Anderson et al. 2013; Herrmann et al. 2013; Russell et al. 2008; Subiaul et al. 2008). However, these studies often lack ecological validity, as non-human primates do not typically interact with humans. Also, while some of these animals were enculturated (i.e., raised in human environments), their natural social interactions in the wild are exclusively between conspecifics, making the findings less generalisable. To address this gap, one study examined Asian elephants (*Elephas maximus*), a species with a 4,000-year history of being tamed to live alongside humans, but did not find evidence that elephants could form reputations of humans (Jim et al. 2021).

Domesticated animals have been selectively bred to pay attention to and interact with humans, making social information about humans more relevant to them (Silver et al. 2021). Research on cats (*Felis silvetris catus*) has not found evidence for reputation formation abilities. Leete et al. (2020) found that cats were neither faster to approach nor spent more time with a friendly human compared to an aggressive human, regardless of whether it was based on direct or indirect experience. Similarly, Chijiiwa et al. (2021) found that cats did not show a significant preference for a human who was helpful to their owner over an unhelpful one. These negative findings could be explained in two ways, which are not mutually exclusive: first, domestic cats descend from solitary animals that typically have minimal social contact, except during the reproductive period (Salamon et al. 2023). Second, cats were not selected for cooperation with humans but rather domesticated for their largely independent role in controlling rodent pests (Chijiiwa et al. 2021). For these reasons, cats may not have evolved the ability to form reputations of others.

In contrast, horses (*Equus caballus*) are group-living animals that have been selectively bred for cooperation with humans, such as in dressage or working as police horses. Research suggests that horses can distinguish between humans based on qualities such as friendliness (Sankey et al. 2010) and skilfulness (Sugimoto and Hirata 2024) after direct interaction. In a study by Trösch et al. (2020), horses watched videos of one human interacting ‘positively’ and another ‘negatively’ with a conspecific; they found that horses touched the negative partner significantly more than the positive partner afterwards, which the authors interpreted as an appeasement behaviour. Interestingly, Krueger et al. (2025) found that horses significantly changed their preference for a feeding location after observing human-human demonstrations, suggesting a capacity for eavesdropping. However, Sugimoto and Hirata (2023) found no such effect, as horses showed no preference for a helper over a non-helper after observing third-party human interactions. These mixed findings suggest that eavesdropping may be more cognitively demanding for horses than direct reputation formation.

Dogs (*Canis lupus familiaris*) are even more cooperative with humans than horses; they were the first species to be domesticated, approximately 23,000 years ago (Perri et al. 2021), and have since been artificially selected to exhibit explicitly desired traits (Udell et al. 2010). Furthermore, dogs are descended from wolves, a highly cooperative, group-living species (Mech and Boitani 2003), which may explain their strong cooperative behaviour with humans, as proposed by the *Canine Cooperation Hypothesis* (Range and Virányi 2015). Throughout much of their domestication history, dogs have lived alongside humans and relied on them for valuable resources like food and shelter (Freidin et al. 2013). This reliance extends to free-ranging dogs, which comprise approximately 80% of the global dog population (Hansen Wheat and Wynne 2025). These factors suggest that the ability to form reputations of humans and use this information to choose the best partners is beneficial for all dogs, regardless of their life experiences.

Dogs may also become more skilled at socially evaluating humans during ontogeny through consistent exposure to and interaction with them (Riedel et al. 2008). For instance, Carballo et al. (2015; Experiment 1) demonstrated that dogs learnt to develop a preference for the generous partner over the selfish partner after repeated interactions. Additionally, Carballo et al. (2017) tested puppies living in families and adult dogs with differing levels of experience with humans. Their findings revealed that adult dogs living in families and in the shelter significantly preferred the generous partner over the selfish partner, whereas puppies did not form a preference. They concluded that experience with humans (in years) may be more important than the quality of the experience (living with a family vs. in a shelter).

For the reasons mentioned above, it seems plausible that dogs can at least form reputations of humans through direct experience, but not all studies have found evidence to support this hypothesis. Several studies have shown that dogs significantly preferred a ‘positive’ partner, such as a generous (Carballo et al. 2015, 2017), competent (Chijiiwa et al. 2022), cooperative (Heberlein et al. 2016, 2017), or nice (Nitzschner et al. 2012; Experiment 1) individual over a ‘negative’ partner, such as one who is selfish, incompetent, competitive, or ignoring. However, other studies have found that dogs do not discriminate between humans based on qualities such as skilfulness or friendliness (Piotti et al. 2017), cooperativeness (McGetrick et al. 2021), generosity (Bray et al. 2014; Jim et al. 2022), or differing intentions (Völter et al. 2023).

The results regarding eavesdropping are even more mixed and difficult to interpret. Early studies found that dogs significantly preferred a generous partner over a selfish partner after observing third-party interactions between humans (Kundey et al. 2011; Marshall-Pescini et al. 2011). However, later studies revealed that these positive results could be explained by local enhancement (Freidin et al. 2013; Nitzschner et al. 2014; Jim et al. 2020). Additionally, Chijiiwa et al. (2015) found that dogs showed a negativity bias in a helping situation (Baumeister et al. 2001), as they did not discriminate between a helpful person and a neutral person, but significantly avoided an unhelpful person compared to a neutral one. Negative information is often prioritised over positive information in a variety of psychological situations and tasks, and this tendency is present early in development, serving critical evolutionarily adaptive functions (Vaish et al. 2008). A limitation of these studies is that the dogs may not have paid attention to the third-party interactions, which were between two humans and not directly relevant to them. Therefore, using third-party interactions between conspecifics and humans would improve the experimental design and potentially allow animals to demonstrate their eavesdropping skills more accurately.

Only a few studies have investigated eavesdropping in dogs using third-party interactions between humans and conspecifics. Rooney and Bradshaw (2006) found that dogs significantly preferred to approach the winner of a tug-of-war game, but Nitzschner et al. (2012; Experiment 2) found that dogs did not prefer an experimenter who was nice to another dog compared to an experimenter who ignored the dog. Moreover, Jim et al. (2022) found that pack-living dogs and wolves, raised in the same environment at the Wolf Science Center (WSC), did not significantly prefer the generous partner over the selfish partner in a food-giving situation after indirect or direct experience. As mentioned previously, ontogeny is a potentially important factor for reputation formation. The WSC animals have more limited experience with humans and do not interact with them in the typical ways that pet dogs do, which may explain discrepancies in the results. Therefore, in the current study, we conducted a similar experiment to Jim et al. (2022), but with pet dogs. Additionally, we assessed the role of age as a proxy for experience, as cognitive abilities change across the lifespan in dogs (Sanches et al. 2022).

This study had two aims: first, we tested whether dogs can form reputations of humans using human-animal interactions in a food-giving situation. Our first hypothesis posits that dogs can socially evaluate humans after direct and indirect experience. We predicted that dogs would significantly prefer the generous partner over the selfish partner, with stronger results for direct experience, as eavesdropping is more cognitively complex. The second aim was to test whether ontogeny affects dogs’ ability to form reputations of humans. Our second hypothesis posits that dogs’ ability to form reputations improves with increased experience with humans. Because age positively correlates with experience, we predicted that older dogs would outperform younger dogs.

## Methods

### Ethical statement

This study was approved by the Ethics and Animal Welfare Committee of the University of Veterinary Medicine Vienna (ETK-023/02/2021). Written informed consent was obtained from all dog owners for their participation in the study and from the individuals in Supplementary Videos 1 and 2 for the publication of photographs and videos containing their images.

### Subjects

44 pet dogs (22 males and 22 females) aged between 1 and 12 years old (*M* = 5.95, *SD* = 3.45) participated in the study (Table S1). They were recruited from a database of owners who volunteered to participate in behavioural studies at the Clever Dog Lab. Four dogs who were not motivated to participate in the study were excluded, resulting in a final sample of 40 dogs (20 males and 20 females, *M* = 5.95, *SD* = 3.40). No breeds were excluded.

### Experimental design

To test the effect of age, we classified dogs into three age groups: 1–3 years as ‘young’ (early adulthood), 4–7 years as ‘adult’ (middle age and late adulthood), and 8–12 years as ‘senior’ (senior and geriatric). These age groups were based on previous papers on ageing in dogs: Wallis et al. (2020) categorised ‘early adulthood’ as > 1–3 years and ‘middle age’ as > 3–6 years; Chapagain et al. (2017) categorised ‘late adulthood’ as > 6–8 years, ‘senior’ as > 8–10 years, and ‘geriatric’ as ≥ 10 years. Our sample was fairly balanced in each age group, with 11 dogs in ‘young’, 15 dogs in ‘adult’, and 14 dogs in ‘senior’.

There were three experimental conditions to test for reputation formation:

1. Eavesdropping (*n* = 20): the subject observed two humans (henceforth ‘partners’) interact with a dog (henceforth ‘dog demonstrator’) to test for indirect reputation formation (i.e., eavesdropping). This condition consisted of two sessions, each with a single trial. Conducting more than one trial per session was avoided to prevent immediate influence from direct experience with the partners, which could confound the assessment of eavesdropping. However, we argue that the single trial in Session 2 was still based on observation rather than the brief interaction in Session 1, as the sessions were separated by several days.
2. Control (*n* = 20): the subject observed the humans perform the same actions as in the eavesdropping condition but without a dog demonstrator being present. This condition was conducted to determine whether the dogs’ responses were due to the interactions between the humans and the dog demonstrator, or if the humans’ actions alone were sufficient to allow a discrimination between them, should eavesdropping be observed in the eavesdropping condition. As with the eavesdropping condition, this condition also consisted of two sessions, each with a single trial.
3. Direct experience (*n* = 40): to test for direct reputation formation, the humans directly interacted with the subject. This condition consisted of a single session with 12 trials.

Dogs were assigned to either the eavesdropping condition or the control condition and matched for age and sex (Table S1). All dogs also participated in the direct experience condition, and the order of conditions was counterbalanced: half of the dogs completed the eavesdropping/control condition first, while the other half completed the direct experience condition first.

In total, 12 women, all unfamiliar to the dogs, volunteered to act as human partners in the study. Pairs of partners remained stable within each condition, but each dog encountered two different pairs of partners (e.g., partners A and B in the first condition and partners C and D in the second condition). During the experiment, one partner was dressed in white and the other in black. The roles and clothing colours were randomised and counterbalanced between subjects but remained fixed within subjects to avoid confusion and help the dogs to better distinguish between the individuals playing the different roles. For example, one subject might have had the generous partner in black and the selfish partner in white in both conditions, though different individuals acted as the partners in each condition. Each partner wore a hip bag containing 18 small pieces of sausage, but only the generous partner used them to feed the dog.

There were two dog demonstrators who interacted with the partners in the observation phase for the eavesdropping group: a neutered male dog (Jasper, 11 years, Labradoodle) served as the demonstrator for female subjects, and a spayed female dog (Emmi, 4 years, Labrador) served as the demonstrator for male subjects. Dogs of the opposite sex were chosen as demonstrators to reduce dominance behaviour and to ensure that subjects would pay more attention to the demonstrator.

### Experimental setup

The experiment was conducted in the outdoor test enclosure of the Clever Dog Lab based at the University of Veterinary Medicine Vienna campus. A chair was placed in the centre of the enclosure, where the dog owner sat during the experiment, and there was a water bowl for the dog next to the chair. The partners stood behind a divider (175 × 180 cm) outside of the enclosure so that the subject could not see them and be distracted in the observation phase. A crate was placed outside the enclosure behind the divider, and the dog demonstrator was placed inside it after the observation phase to avoid disturbing the subject during the test phase.

**Fig. 1:**
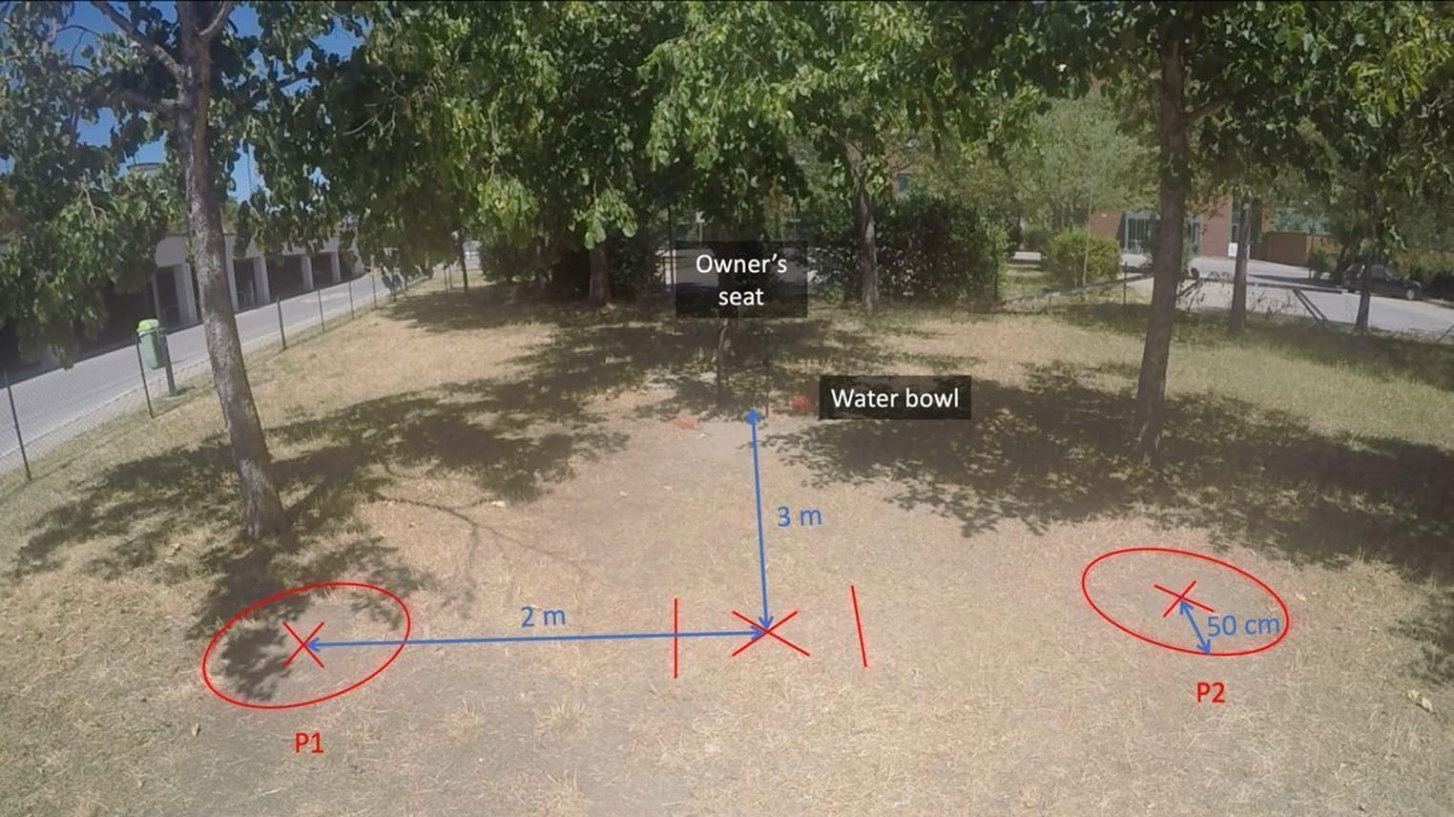
Wide-angle photo of the experimental setup

We used spray paint on the grass to mark where individuals would stand during the experiment. A cross was marked 3 m away from the owner’s seat and two crosses were marked 2 m perpendicular from the central cross. A circle with a radius of 50 cm was marked around the left (P1) and right (P2) crosses (Fig. 1). A camera (GoPro Hero 4 Black) was placed on a tripod in the enclosure and filmed the whole experiment.

### Procedure

**Fig. 2:**
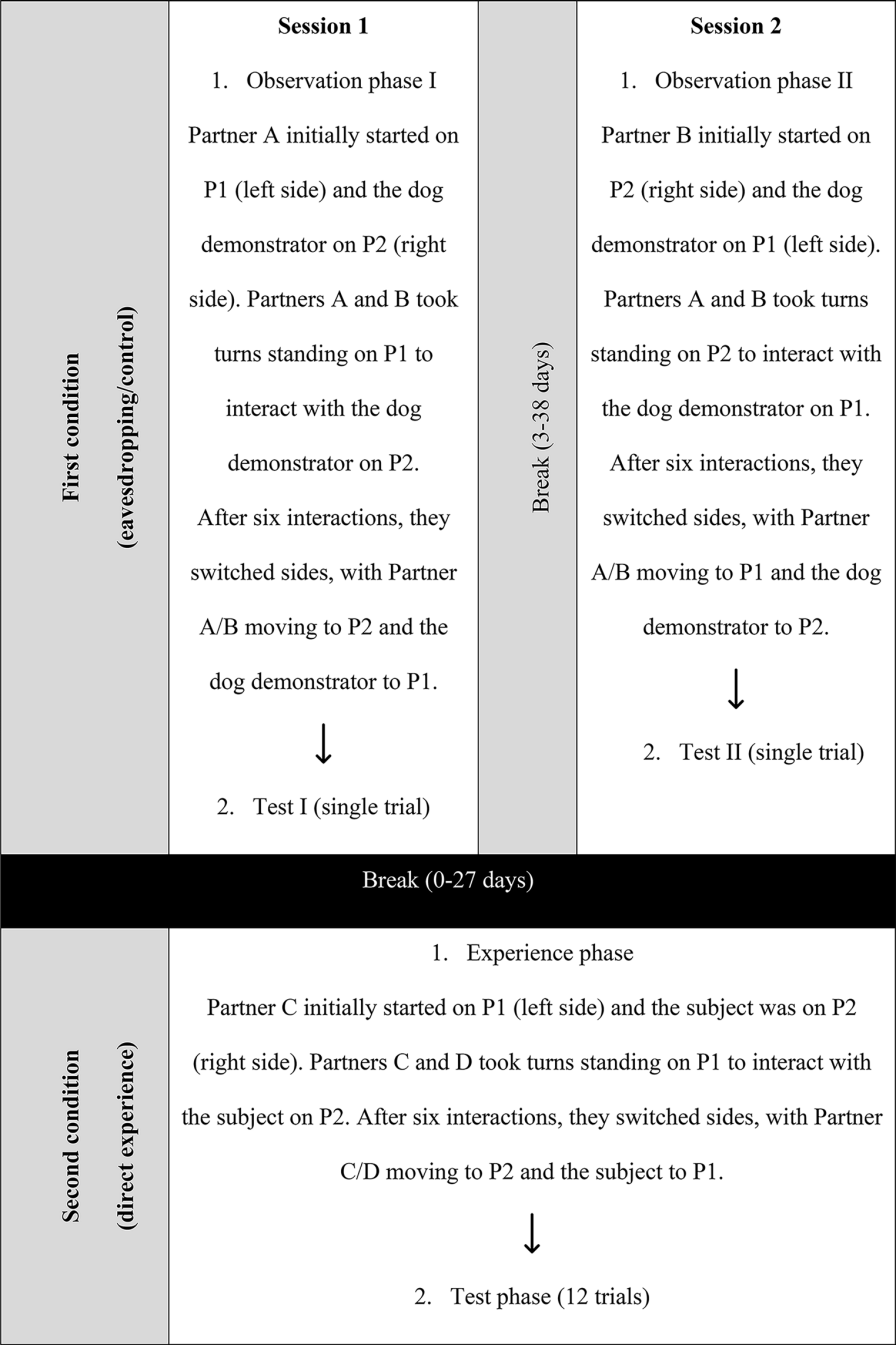
Flowchart illustrating an example of the procedure for one subject

The study was conducted from May to August 2021 and generally followed the procedure outlined in Jim et al. (2022) (see Fig. 2 for an overview).

#### Eavesdropping/control condition

This condition comprised two sessions and there was a break of 3–38 days between sessions. The first session began with a *habituation phase*, where the owner and main experimenter took the subject and the dog demonstrator on a short walk around the university campus (5–10 min) to ensure that they tolerated each other and could then focus during the experiment (if the dogs had been uncomfortable with each other, the experiment would have been terminated, but termination never occurred). The subject and the owner then entered the enclosure, and the subject could explore it freely for five minutes while the main experimenter explained the procedure to the owner.

Before the main procedure, a *baseline* was conducted to determine whether dogs, as a group, exhibited a preference for one partner over the other before observing any third-party interactions. The owner sat with their dog between their legs, holding the dog by the collar, and remained blindfolded throughout the experiment to prevent them from influencing the dog’s behaviour. The partners entered the enclosure and stood on P1 and P2 (randomised and counterbalanced across subjects) without making eye contact with the dog. Each person held a piece of sausage in their hand, keeping their arms relaxed by their sides so it was not obvious to the dog that they were holding food, but allowing them to immediately reward the dog upon approach. When the main experimenter said “ok”, she started the timer for one minute and the owner let go of the dog’s collar. The owner could give a short prompt if the dog did not move by themselves, such as a gentle nudge or saying “ok” to indicate to the dog that they were free to move, but was instructed not to gesture in a specific direction. If the subject walked towards a partner and at least one front paw was within or on the circle marked on the floor whilst looking at her, this behaviour was considered as a choice and the partner fed the dog (see Table 1). After the subject ate the food, the other partner who was not chosen called the subject to get their attention and fed the dog too to ensure they did not develop a preference for one partner. If the subject did not approach either partner after one minute, it was considered a ‘no choice’ (though this never occurred). Then, the main experimenter asked the owner to call the dog back. The owner remained seated, holding their dog by the collar, and the partners left the enclosure. The baseline ended after this single trial (Supplementary Video 1).

The main procedure comprised two phases:

1. Observation phase.

In the *eavesdropping condition*, the main experimenter entered the enclosure with the dog demonstrator on a short lead and stood on P1 or P2 (randomised and counterbalanced across subjects). Then, one partner entered the enclosure and walked to the opposite standing point, holding a piece of sausage in her hand. The main experimenter walked alongside the dog demonstrator toward the partner but then stayed behind, allowing the dog demonstrator to approach the partner alone. When the dog demonstrator reached the partner, she raised her arm to show the food. The subject witnessed one of the following scenarios, depending on which partner the dog demonstrator interacted with:

- Generous: The partner said, “here you go!” or “du kannst es haben!” in a friendly tone and fed the dog demonstrator (the partners spoke in their preferred language to ensure their behaviour was natural). After the dog demonstrator had eaten the food, the main experimenter walked the dog demonstrator back to the starting point, while the partner left the enclosure.
- Selfish: The partner said, “you can’t have it!” or “du kriegst es nicht!” in an unfriendly tone, crossed her arms, and turned away, keeping the food in her hand. After the interaction ended a few seconds later, the main experimenter walked the dog demonstrator back to the starting point, and the partner left the enclosure.

This phase was identical in the *control condition*, with one difference: the main experimenter and dog demonstrator were absent. Thus, the subject observed the partners ‘interact’ with an invisible dog in the same way as in the scenarios described above (Supplementary Material 1).

The procedure was repeated three times per partner (e.g., six interactions in total with the main experimenter and dog demonstrator standing on P1 and the partners standing on P2). Then, the main experimenter and dog demonstrator swapped sides, and the entire procedure was repeated (e.g., six interactions with the main experimenter and dog demonstrator now standing on P2 and the partners standing on P1), resulting in 12 interactions in total during the observation phase. After these interactions, the main experimenter and the dog demonstrator left the enclosure, the dog demonstrator was placed in the crate, and the test phase immediately followed.

2. Test phase.

The test phase consisted of a single trial and was identical in both the eavesdropping and control conditions. Both partners entered the enclosure, with one standing on P1 and the other on P2 (randomised and counterbalanced across subjects), neither holding food, standing still and looking ahead without making eye contact with the dog. When the main experimenter said “ok”, she started the 15-second timer, and the owner released the dog’s collar. As in the baseline, the owner could give a short prompt if the dog did not move by themselves. During this time, the dog could act freely while the partners remained still and did not react to the dog. The partners acted neutrally and did not have food, ensuring that they could not display generous or selfish behaviour; this neutral setup was intended to prevent the formation of reputations based on direct experience in the first test trial, which could have influenced their behaviour in the second test trial. At the end of this single trial, the main experimenter said “stop” to indicate to the owner to call the dog back. The owner remained seated, holding their dog by the collar, and the partners left the enclosure, which concluded the session.

Session 2 (conducted 3–38 days later) was identical to Session 1, with some minor changes. There was no habituation phase or baseline, so it started immediately with the observation phase. The order of the partners and the side they stood on first was counterbalanced across sessions (e.g., if the selfish partner started and stood on P1 in Session 1, then the generous partner started and stood on P2 in Session 2). The positions of the generous and selfish partner were also counterbalanced across sessions in the test phase. Again, there was only one 15-second trial (identical to Session 1), and this single trial concluded the session.

#### Direct experience condition

Prior to testing, the subject was allowed to explore the enclosure freely for approximately five minutes while the main experimenter explained the procedure to the owner. This condition comprised a single session and no baseline was conducted. Hence, the procedure comprised two phases:

1. Experience phase.

The experience phase was identical to the observation phase in the eavesdropping/control condition, except that the owner replaced the main experimenter and the subject replaced the dog demonstrator. The owner acted as the main experimenter to prevent potential anxiety from separation, which could have affected the dog’s focus during testing. To ensure consistency across subjects, we used this approach with all dogs. This approach also aligns with the procedure in Jim et al. (2022), where a familiar trainer held the lead, maintaining a comparable setup. Thus, the owner entered the enclosure with the subject on a short lead and stood on P1 or P2 (randomised and counterbalanced across subjects). One partner then entered the enclosure, walked to the opposite standing point, and interacted with the subject in the same way they would with the dog demonstrator in the eavesdropping/control condition. This procedure was repeated three times per partner, resulting in six interactions with the owner and subject standing at P1 and the partners at P2. The owner and subject then swapped sides, and the procedure was repeated (i.e., six interactions with the owner and subject now standing at P2 and the partners at P1). After these 12 interactions, the test phase immediately followed.

2. Test phase.

Before the first trial, the owner exchanged the short lead for a 10-metre training lead and sat with their dog between their legs, holding the dog by the collar. The long lead allowed the dog to move freely around the enclosure during the trial but facilitated recall at the end of each trial.

Both partners entered the enclosure, with one standing on P1 and the other on P2, each holding a piece of sausage in their hand, with their arms relaxed by their sides. When the main experimenter said “ok”, indicating the owner to release their dog, she started the timer for 15 s. Simultaneously, the partners raised their hands to show the food to the dog, standing still and looking ahead without making eye contact with the dog. As in the test phase of the eavesdropping/control condition, the owner could give a short prompt if the dog did not move by themselves.

If the subject walked towards a partner and at least one front paw was within or on the circle marked on the floor whilst looking at her, this behaviour was considered as a choice (see Table 1). The partner then acted the same way as during the experience phase (i.e., the generous partner fed the subject, while the selfish partner did not). At the same time, the other partner moved her hands to her chest to prevent the dog from taking the food, and both partners ignored the dog for the remainder of the trial. If the dog did not approach either partner during the 15 s, it was considered a ‘no choice’.

At the end of the trial, the experimenter said “stop” to indicate to the owner to call the dog back and hold them by the collar. If the generous partner was chosen, she rebaited herself by taking another piece of sausage from her hip bag, and the selfish partner performed the same action simultaneously to control for stimulus enhancement. The partners’ positions were semi-randomised to ensure they did not stay in the same position more than twice consecutively. The main experimenter used hand signals to silently instruct the partners to either remain in their positions or swap places during each trial; this method was used to minimise the likelihood of the blindfolded owner becoming aware of the partners’ positions, as having such knowledge could allow them to influence the dog’s behaviour. After these 12 trials, this concluded the session (Supplementary Video 2).

As stated previously, dogs were tested in the eavesdropping/control condition and direct experience condition in a counterbalanced order. If dogs completed the direct experience condition first, there was a break of 2–26 days between this condition and Session 1 of the eavesdropping/control condition. However, if dogs completed the eavesdropping/control condition first, we allowed them to participate in the direct experience condition on the same day after a break of 15–30 min due to unavoidable logistical constraints (e.g., coordinating schedules with owners and partners). As a result, the interval between Session 2 of the eavesdropping/control condition and the direct experience condition ranged from 0 to 27 days. Importantly, the dogs interacted with two different sets of partners in the two conditions they experienced.

### Behavioural analysis

The videos were coded on Loopy (http://loopb.io, Loopbio Gmbh, Vienna, Austria). We coded the dog’s choice in the baseline and test trials. The trial started when the experimenter said “ok” and stopped when the experimenter said “stop” after 15 s. We live coded the dog’s first choice as a binomial variable (generous or selfish). If the dog did not make a choice within the trial, then it was a ‘no choice’ response and coded as NA and removed from the analysis.

In the test trials of the direct experience condition, a choice was only coded if the partner reacted to the subject (i.e., the generous partner fed the dog and the selfish partner turned away). To maintain consistency with the experience phase—where the dog approached only one partner at a time and immediately experienced that partner’s reaction—only the first partner the dog approached reacted to them during the test trial. Interobserver reliability was not analysed for choice because it was clear which partner the dog approached first and we could review the video footage if necessary.

We also coded how much time the subject spent exhibiting affiliative behaviours towards each partner within the 15-second trial (see Table 1 for definitions). 20% of the videos were randomly selected for interobserver reliability, which were coded by KB and EBM and analysed with R (v4.4.0; R Core Team 2024) using the Intraclass Correlation Coefficient from the R package “irr” (v0.84.1; Gamer et al. 2019). Inter-rater agreement was excellent (ICC (two-way, agreement) = 0.993, *F* = 308, *p* <.001). KB and EBM then coded half of the remaining videos each.

**Table 1:** Definitions of coded behaviours

Behaviour	Definition
**Choice (generous / selfish / NA)** (Event)	At least one of the subject’s front paws was within or on the circle (radius of 50 cm) marked on the floor whilst looking at the partner.
**Affiliative behaviours towards partner (generous and selfish were coded separately)** (Duration in seconds)	• At least one of the subject’s paws was within or on the circle (radius of 50 cm) marked on the floor. • The subject moved their paw outside of the circle but stayed stationary near the partner until their whole body moved away. • The subject stepped out of the circle but continued to look at the partner until they looked away. • The subject was jumping on the partner and their paws left the circle. • If the dog walked past the partner and entered the circle but did not stop or look at her, this behaviour was not coded.

### Statistical analysis

All analyses were performed using R in RStudio (v2024.04.0 + 735; Posit team 2024) and graphs were created using the R packages “ggplot2” (v3.5.1; Wickham 2016) and “ggeffects” (v1.7.0; Lüdecke 2018).

#### Baseline

First, we analysed whether dogs, as a group, preferred one partner over the other before observing any third-party interactions. To assess this preference, we conducted an exact binomial test to compare the proportion of dogs that chose one partner with the proportion expected by chance (0.5). However, even if the whole sample of dogs did not have a significant preference for one partner over the other, it is possible that a subsample in the different age groups or conditions (eavesdropping or control only) could have had a bias. Therefore, we conducted a generalised linear model (GLM) with a binomial error distribution using the function ‘glm’ to test whether age group and condition (both included as categorical predictors) influenced dogs’ preference for one partner over the other. In these analyses, the individual who had been assigned the generous role for the main procedure was coded as a ‘success’ in the response variable. This model consisted of the single trial in the baseline from the 40 dogs.

#### Test phase

##### Eavesdropping vs. control condition

***Partner choice*** To examine whether the proportion of choices for the generous partner depended on age group (young, adult, senior) and/or condition (eavesdropping vs. control), we conducted a generalised linear mixed-effects model (GLMM) with a binomial error structure and logit link function (McCullagh and Nelder 1989), using the function ‘glmer’ from the R package “lme4” (v1.1.33; Bates et al. 2015). Age group and condition, along with their two-way interaction, were included as categorical predictors. Trial number (1 or 2) and condition order (i.e., whether dogs experienced the control/eavesdropping condition before or after the direct experience condition) were added as control variables. Trial number was treated as a factor with two levels, while condition order was treated as a continuous fixed effect. Subject ID was included as a random effect to account for repeated measures. After discarding six trials where dogs did not make any choice, we analysed data from 74 trials involving 39 dogs (one dog, Mozart, did not choose a partner in either of the two trials across both conditions).

***Time spent exhibiting affiliative behaviours towards the generous partner*** We also tested whether dogs spent more time with the generous partner using a GLMM with a beta distribution, implemented with the R package “glmmTMB” (v1.1.7; Brooks et al. 2017). The predictors, their two-way interaction, control variables, and random effect were identical to those used in the binomial GLMM described above. The response variable was the proportion of time spent exhibiting affiliative behaviours towards the generous partner during each trial within each condition, calculated as the time exhibiting affiliative behaviours towards generous partner / (time exhibiting affiliative behaviours towards generous + selfish partner). After discarding eight trials where dogs did not spend any time exhibiting affiliative behaviours towards either partner, we analysed data from 72 trials involving 39 dogs (one dog, Mozart, did not approach a partner in either of the two trials across both conditions) to calculate proportions by trial. Lastly, as beta GLMMs cannot handle 0s and 1s, we transformed the response variable following the formula suggested by Smithson and Verkuilen (2006).

##### Direct experience condition

***Partner choice*** We analysed whether dogs preferred the generous partner after the experience phase at both an individual level and group level, using the proportion of choices for the generous partner as the response variable. This was calculated as the number of choices for the generous partner / (number of choices for the generous + selfish partner). At the individual level, we conducted exact binomial tests to compare this proportion against the value expected by chance (0.5). At the group level, we conducted a GLMM with a binomial error structure and logit link function to examine whether the proportion of choices for the generous partner varied by age group (young, adult, senior) and/or trial number (1–12, as more trials equated to more experience and opportunities to learn). Age group, trial number, and their two-way interaction were included as predictors. Condition order was included as a continuous fixed-effect control variable. Subject ID was included as a random effect to account for repeated measures. After discarding 38 trials where dogs did not make any choice, we analysed data from 442 trials involving the 40 dogs.

***Time spent exhibiting affiliative behaviours towards the generous partner*** We tested whether dogs spent more time with the generous partner using a GLMM with a beta distribution. The predictors, their two-way interaction, control variable and random effect were identical to those used in the corresponding binomial GLMM described above. We calculated the proportion of time spent exhibiting affiliative behaviours towards the generous partner in each trial as the response variable. After discarding 100 trials where dogs did not spend any time exhibiting affiliative behaviours towards either partner, we analysed data from 380 trials involving the 40 dogs to calculate proportions by trial. Lastly, as beta GLMMs cannot handle 0s and 1s, we transformed the response variable following the formula suggested by Smithson and Verkuilen (2006).

We followed the same procedure to fit the GLMMs: we z-transformed condition order (a continuous fixed effect) to a mean of 0 and standard deviation of 1. To avoid inflated type I error caused by multiple testing (Forstmeier and Schielzeth 2011), we used a full-null model approach, comparing the significance of full models by means of a likelihood ratio test (Dobson and Barnett 2018) using the R function ‘anova’ with the argument test set to “Chisq”, with null models lacking the predictors but otherwise identical to their respective full model. *p* values for individual effects were based on likelihood ratio tests comparing the full model with reduced models lacking each term. If the model included an interaction that was not significant, we removed the interaction from the model and fitted a reduced model including only the single terms to ease interpretation of the single term’s estimations. We calculated profile likelihood confidence intervals based on the log-likelihood function.

We evaluated the quality of the models following a series of steps. We checked for model stability for the baseline GLM by means of dfbeta-values, and in the case of the GLMMs by excluding subjects one at a time from the data and comparing model estimates derived for these subsets with the main model, using a R function provided by Mundry (2023). All models were fairly stable. To check for collinearity problems, we inspected variance inflation factors (VIF) (Field 2005) from a linear model with the same terms as the full model excluding the interaction and random effects using the function ‘vif’ in the R package “car” (v3.1.2; Fox and Weisberg 2019). We did not detect any collinearity issues (all VIF close to 1). We checked whether there was a problem with overdispersion in the two beta GLMMs and found the dispersion parameter of both to be acceptable (eavesdropping vs. control condition model = 1.155; direct experience condition model = 1.065). We inspected the histograms of the Best Linear Unbiased Predictors (BLUPs) to confirm that there was no deviation from a normal distribution.

## Results

### Baseline

The exact binomial test revealed that 20 dogs chose one partner and 20 chose the other partner; this proportion does not deviate from chance (binomial probability, estimate = 0.5, 95% CI = 0.338–0.662, *p* = 1). Thus, dogs, as a group, did not prefer one partner over the other before observing any third-party interactions. Furthermore, the binomial GLM revealed that the probability of choosing one of the two partners was not predicted by age group or condition (full-null model comparison, *deviance* = 5.796, *df* = 3, *p* =.122; Table S2). These results indicate that dogs did not have a significant preference for one partner over the other before observing any third-party interactions in any subgroup.

### Test phase

#### Eavesdropping vs. control condition

##### Partner choice

**Fig. 3:**
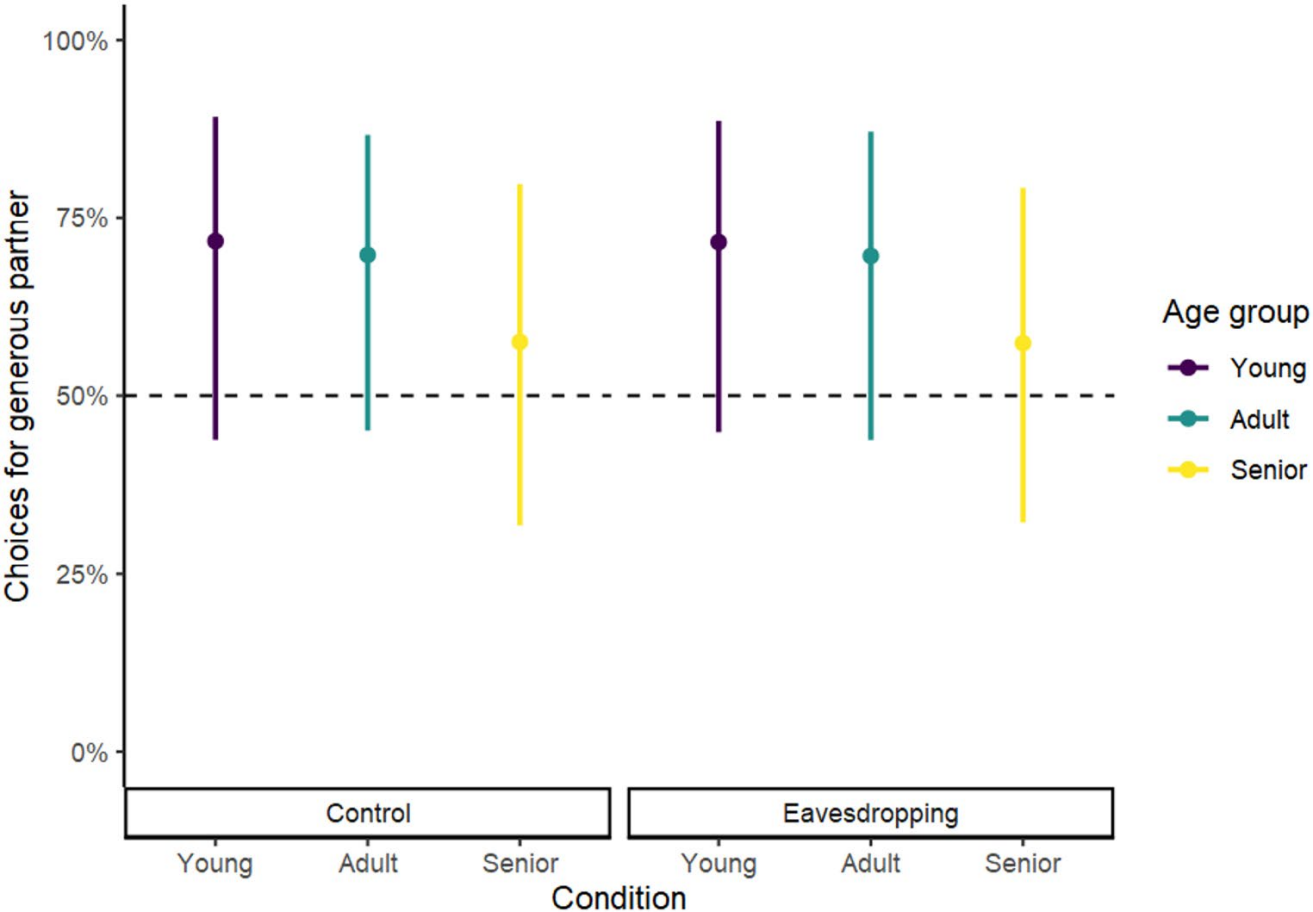
Estimates (circles) and confidence intervals (lines) indicating the percentage of choices for the generous partner by age group and condition. The horizontal dotted line represents the chance level (50%)

The binomial GLMM revealed that the full model including age group, condition, and its interaction was not better than the null model lacking these variables (full-null model comparison, χ^2^ = 1.344, *df* = 5, *p* =.930; Table S3). These results indicate that neither age group nor condition influenced the likelihood of choosing the generous partner. Also, as shown in Fig. 3, all model estimates and confidence intervals include the chance level of 50%, which means that none of the age groups chose the generous partner above chance level in either condition.

##### Time spent exhibiting affiliative behaviours towards the generous partner

**Fig. 4:**
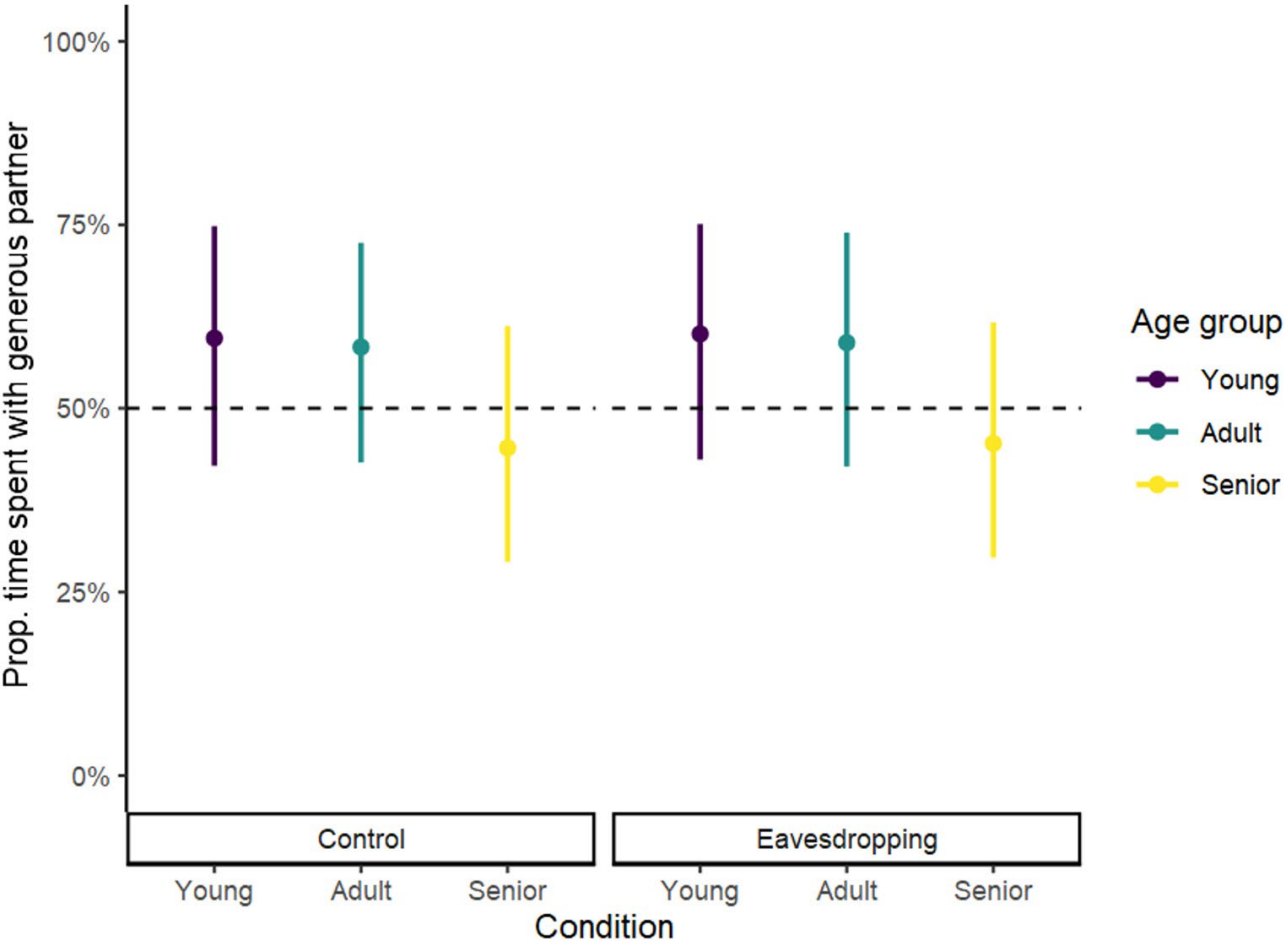
Estimates (circles) and confidence intervals (lines) indicating the proportion of time exhibiting affiliative behaviours towards the generous partner by age group and condition. The horizontal dotted line represents the chance level (50%)

The beta GLMM revealed that the amount of time that dogs spent exhibiting affiliative behaviours towards the generous partner was not influenced by age group or condition (full-null model comparison, χ^2^ = 4.016, *df* = 5, *p* =.547; Table S4). Moreover, Fig. 4 shows that all model estimates and confidence intervals include the chance level of 50%, indicating that none of the age groups spent more time with the generous partner than with the selfish partner in either condition.

#### Direct experience condition

##### Partner choice

We analysed whether dogs preferred the generous partner after the experience phase, first at an individual level and then at a group level. The exact binomial tests showed that three dogs out of 40 showed a significant preference for one partner: Amy (11 years, senior) chose the generous partner in 9 out of 9 trials (binomial probability estimate = 1.000, CI = 0.664-1.000, *p* =.004); Dunni (1 year, young) chose the generous partner in 10 out of 12 trials (binomial probability estimate = 0.833, CI = 0.516–0.979, *p* =.039); and Snoopy2 (12 years, senior) chose the generous partner in 1 out of 10 trials (binomial probability estimate = 0.100, CI = 0.003–0.445, *p* =.021). Note that the number of trials varied across animals because trials in which subjects did not make a choice were excluded from the analyses.

**Fig. 5:**
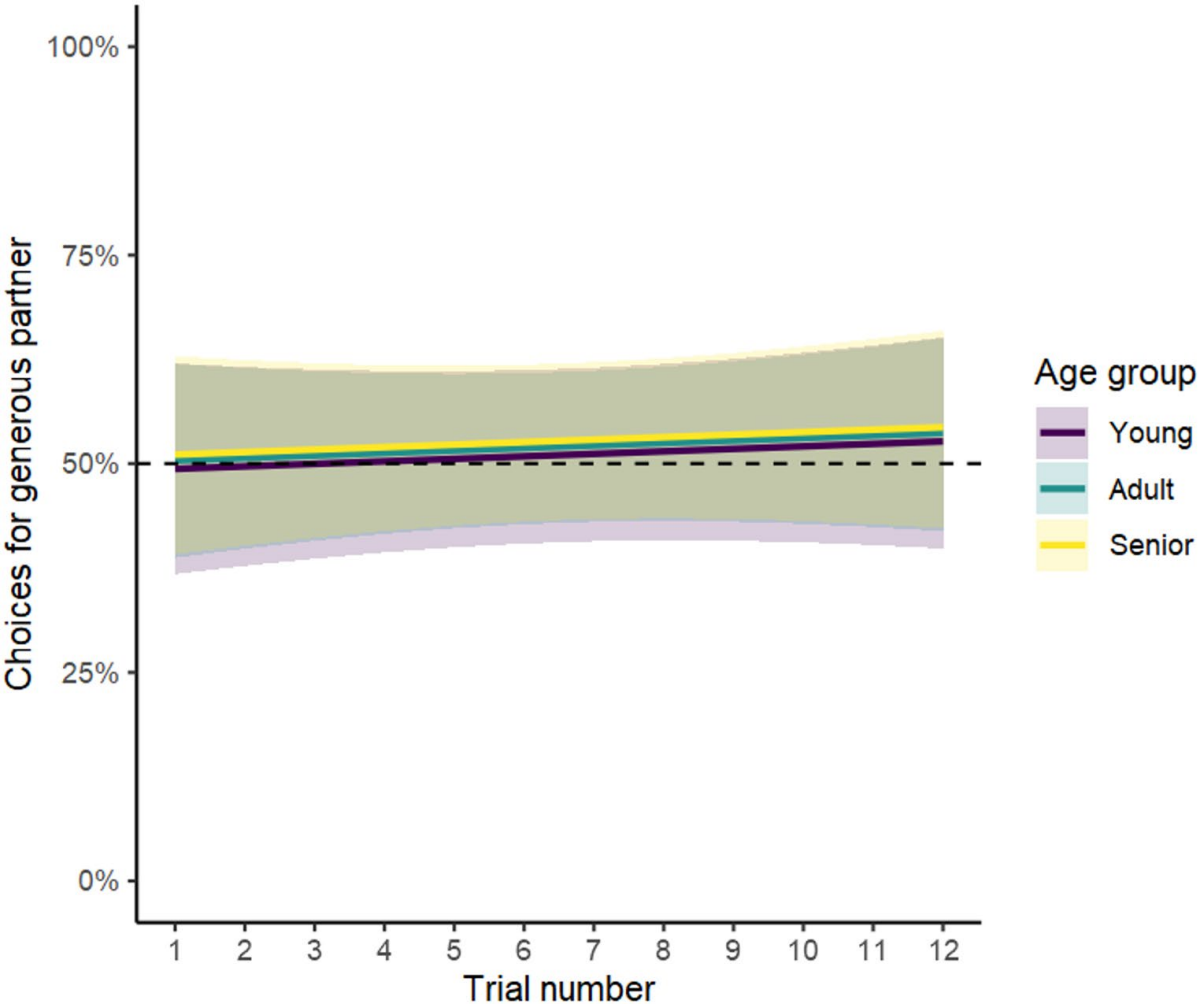
Estimates (lines) and confidence intervals (shaded area) indicating the percentage of choices for the generous partner by age group and trial in the direct condition. The horizontal dotted line represents the chance level (50%)

At the group level, the binomial GLMM revealed that the full model, which included age group, trial number, and their interaction, was not better than the null model lacking these variables in predicting the likelihood of choosing the generous partner (χ^2^ = 1.811, *df* = 5, *p* =.875; Table S5). These results indicate that young, adult, and senior dogs were equally likely to choose the generous partner over the selfish one and did not change their choices with more experience (i.e., across trials). As shown in Fig. 5, all model estimates and confidence intervals include the chance level of 50%, suggesting that dogs’ choices did not deviate from chance.

##### Time spent exhibiting affiliative behaviours towards the generous partner

**Fig. 6:**
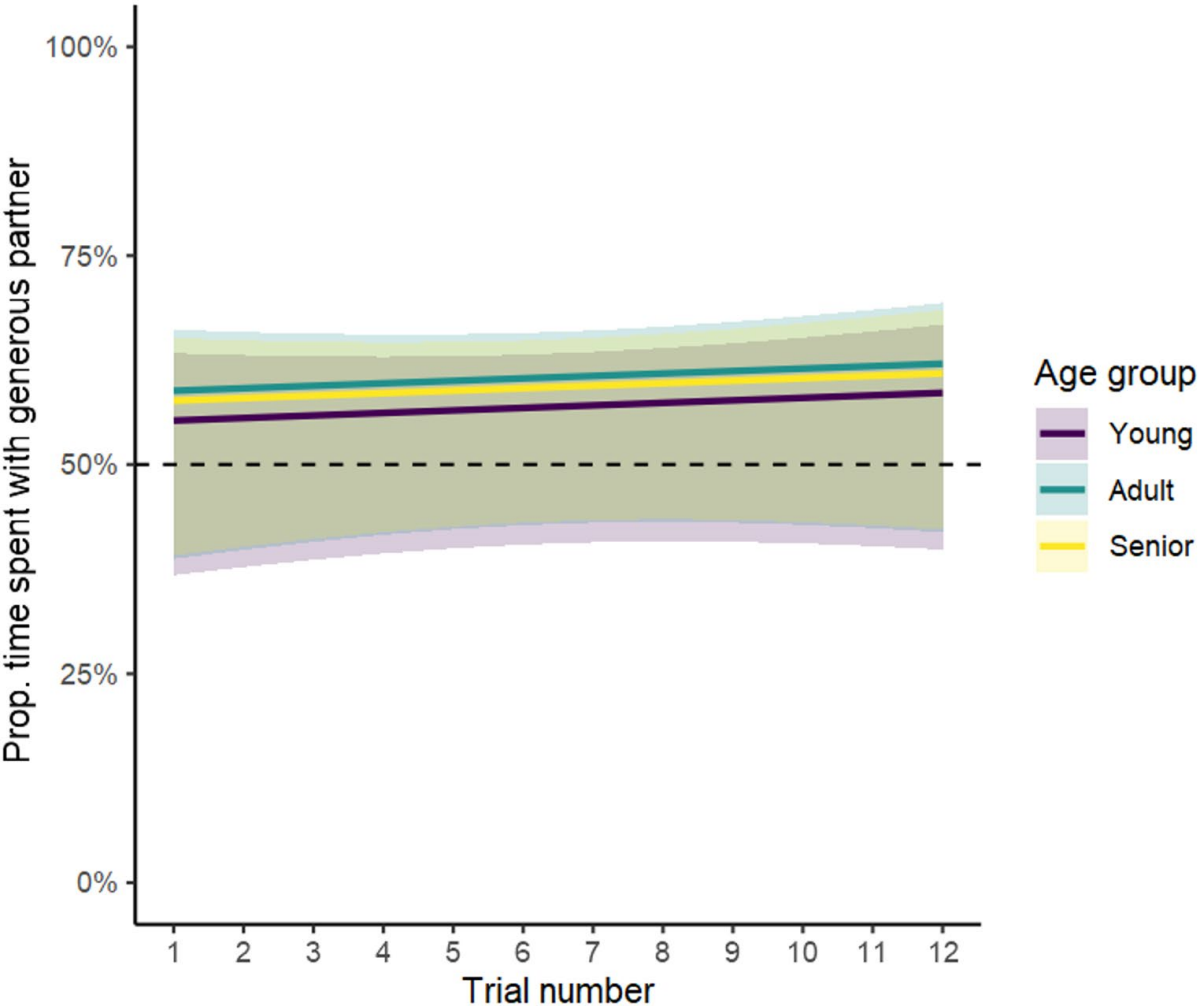
Estimates (lines) and confidence intervals (shaded area) indicating the percentage of time spent with the generous partner by age group and trial. The horizontal dotted line represents the chance level (50%)

The beta GLMM revealed that the amount of time spent exhibiting affiliative behaviours towards the generous partner was not influenced by age group or trial number (full-null model comparison, χ^2^ = 1.291, *df* = 5, *p* =.936; Table S6). Fig. 6 illustrates that all model estimates and confidence intervals include the chance level of 50%, indicating that none of the age groups spent more time with the generous partner than with the selfish partner.

### Colour and side bias

As the results were non-significant, we conducted exact binomial tests to determine whether each subject showed a bias for colour or side when choosing a partner in the test trials. These analyses focused on the direct experience condition, as it was the only condition with enough trials to calculate binomial probabilities (the eavesdropping/control condition included only two trials). The partner wearing black or standing on P1 (left side) was coded as a ‘success’ for colour and side, respectively, and we compared the proportion of trials in which dogs chose this partner with the proportion expected by chance (0.5). Colour and side were always counterbalanced across trials.

Of the 40 dogs, two showed a colour bias, 15 showed a side bias, and one dog showed both a colour and side bias (Table S7). Specifically, one dog preferred the partner wearing black clothes, while another preferred white. Five dogs preferred the partner standing on P1 (left side) and 10 dogs preferred P2 (right side). Since over 37% of the sample showed a side bias, we excluded these 15 dogs and repeated the analyses on choice and affiliative behaviours for both the eavesdropping vs. control condition and the direct experience condition. In these analyses, age was tested as a continuous predictor, which may be more sensitive to developmental changes than treating age as a categorical variable. These follow-up analyses were conducted to clarify whether our findings were strongly influenced by side bias, but the results did not change (Supplementary Material 2).

## Discussion

This study investigated whether pet dogs of different ages can socially evaluate humans after observing them interact with a conspecific and/or after directly interacting with them in a food-giving situation. Reputation formation was assessed through the dogs’ partner choice and time spent exhibiting affiliative behaviours towards each partner. Among the 40 dogs tested, only three (one young and two senior) significantly approached one partner more (two preferred the generous partner and one preferred the selfish partner), all within the direct experience condition. These findings suggest that direct reputation formation may be less cognitively complex than eavesdropping. However, across all age groups, dogs did not significantly choose to approach or spend more time exhibiting affiliative behaviours towards the generous partner compared to the selfish partner, nor did they perform above chance level, after indirect or direct experience with the humans. Thus, our findings do not support either of our hypotheses: (1) that dogs can socially evaluate humans after direct and/or indirect experience, and (2) that dogs’ ability to form reputations improves with increased experience with humans, as assessed by age.

Our results regarding our first hypothesis—that dogs can form reputations of humans through indirect and/or direct experience—contrast with previous studies reporting evidence for both direct (e.g., Nitzschner et al. 2012; Experiment 1; Carballo et al. 2015, 2017; Heberlein et al. 2016, 2017; Chijiiwa et al. 2022) and indirect (e.g., Rooney and Bradshaw 2006; Kundey et al. 2011; Marshall-Pescini et al. 2011; Chijiiwa et al. 2015; Silver et al. 2021) reputation formation in dogs. However, our findings align with others that found no such effect for direct (e.g., Bray et al. 2014; Piotti et al. 2017; McGetrick et al. 2021; Jim et al. 2022; Völter et al. 2023) or indirect (e.g., Nitzschner et al. 2012, 2014; Freidin et al. 2013; Jim et al. 2020, 2022) reputation formation.

Our methods were designed to address procedural limitations in previous studies investigating social evaluation in dogs, which may have contributed to their positive results (e.g., Kundey et al. 2011; Marshall-Pescini et al. 2011; Chijiiwa et al. 2015; Silver et al. 2021). Specifically, these studies did not control for experimenter position, meaning the results may be attributed to local enhancement rather than genuine eavesdropping (Freidin et al. 2013; Nitzschner et al. 2014; Jim et al. 2020). Our study adopted a similar procedure to Jim et al. (2022) and accounted for the critical factors that may have affected results in previous studies, including controlling for stimulus and local enhancement effects. In Jim et al. (2022), we found that pack-living dogs and wolves at the WSC did not significantly prefer the generous partner over the selfish partner in a food-giving situation after indirect or direct experience. The sample in that study comprised only six adult dogs, which greatly limited the power of the statistical analyses. Furthermore, the dogs at the WSC have more limited experience with humans and do not interact with them in the typical way pet dogs do. While the negative findings of Jim et al. (2022) may reflect the distinctive context of WSC dogs and/or statistical limitations, the alignment of our current findings—involving 40 pet dogs with different ontogenic experiences—could suggest a lack of capacity in dogs to form reputations. These results contribute to the growing body of literature suggesting that dogs cannot socially evaluate humans through third party interactions. As direct reputation formation is a prerequisite for eavesdropping, it is unsurprising that we found no evidence of eavesdropping, given that dogs did not demonstrate reputation formation even after direct experience. Nevertheless, the lack of evidence for direct reputation in our study is unexpected and we propose that methodological challenges in experimental design may account for our negative findings, rather than a lack of capacity.

Aware of the possibility that repeated experiences may be necessary for direct reputation formation, we increased the number of interactions from four to six per partner in the observation/experience phase and extended the test phase in the direct experience condition from six to twelve trials, compared to Jim et al. (2022). Despite these adjustments, we still found no evidence of direct reputation formation. In Subiaul et al. (2008), seven chimpanzees (*Pan troglodytes)* underwent extensive training with two human partners to form generous and selfish reputations through direct experience, requiring 32–184 trials before four chimpanzees could reliably choose the familiar generous donor. Even then, one chimpanzee failed to maintain a preference for this donor, while two did not pass criterion training. These findings suggest that even cognitively complex species, such as chimpanzees, may face significant difficulties in forming reputations even through direct experience. A potential solution could be to present fewer interactions spread over multiple sessions before testing, allowing animals repeated experiences without creating a boredom or fatigue effect from experiencing all the repetitions on the same day/session.

Another non-mutually exclusive explanation for our negative results is that dogs may have struggled to differentiate between the partners in this study. Carballo et al. (2015; Experiment 2) found that dogs were faster at discriminating between the partners when they were of different genders. In the current study, the two female partners wore contrasting clothing to make their distinguishing features more salient. However, this visual cue might not have been enough to overcome potential experimental limitations, particularly if the number of interactions remained inadequate. We also cannot rule out the possibility that the dogs did form reputations of the humans but simply failed to demonstrate evidence of this formation during the test phase. Ethical considerations often necessitate using positive interactions, such as food-giving contexts, to ensure animals enjoy participating. Consequently, we expected dogs to prefer the positive partner who was more likely to benefit them, but we might have observed significant results if we had tested for a negativity bias instead.

Lastly, we found that 37% of the sample showed a side bias, with most dogs significantly preferring the partner standing on the right side, and our results did not change after we excluded these dogs. A likely explanation for this bias is that the study was conducted in an outdoor test enclosure during the summer months and the right side of the enclosure provided more shade (Supplementary Video 1). While conducting the study outdoors allowed for comparability with Jim et al. (2022), it also introduced the possibility of environmental distractions; these challenges highlight the importance of conducting experiments within a controlled research laboratory setting to minimise environmental factors that could potentially impact dogs’ performance in such cognitive tasks. That said, the controlled nature of laboratory experiments differs from real-life situations, where such environmental factors are naturally present, but the stakes may be higher, potentially prompting animals to overcome such minor biases.

While these methodological challenges provide insight into potential limitations of our study design, they may not fully explain why the dogs failed to show evidence of reputation formation. Ontogeny may play a role, as pet dogs occupy a unique niche (Hansen Wheat and Wynne 2025) characterised by stable bonds and frequent positive interactions, which may reduce their sensitivity to forming specific preferences based on reputation. First, pet dogs’ exposure to friendly, unfamiliar humans in their daily lives may have made them generally more comfortable approaching the partners indiscriminately in the test phase. Second, persistence is likely to have been rewarded in pet dogs’ past experiences, as refusal or ignoring by humans rarely results in negative consequences. Therefore, they may have been less sensitive to differentiating between the partners’ behaviours in this study. This persistence may also delay the formation of reputations, aligning with findings by Carballo et al. (2015; Experiment 1). Third, pet dogs are generally well-loved and cared for and were not food-deprived before the study, which may have lowered the stakes in our setup and reduced their motivation to form a preference for the generous partner. Alternatively, the mere presence of food may have been overly distracting, as suggested by Nitzschner et al. (2012); the partners raised their hands to show the food, making it visible and salient, which may have overshadowed the individual differences between the two experimenters. However, we maintained this approach to ensure comparability with Jim et al. (2022). Nevertheless, a food-giving situation is likely to be highly relevant and motivating for free-ranging dogs, which could yield highly ecologically valid results, making them promising candidates for future studies.

Finally, our second hypothesis—that experience with humans, as measured by age, would affect dogs’ ability to form reputations—was also not supported. Our results do not align with those of Carballo et al. (2017), the only other study, to our knowledge, that investigated age-related effects on direct reputation formation. In their study, adult dogs (both from families and shelters) developed a preference for the generous partner over the selfish one, while puppies did not.

In conclusion, our study does not provide support that pet dogs, regardless of age, are capable of forming reputations of humans after observing them interact with a conspecific or through direct interactions. Because no evidence of reputation formation was found in any age group, our second hypothesis could not be tested. These findings add to the growing body of literature suggesting that social evaluation is challenging for animals. To further explore how ontogeny may influence this sociocognitive skill, future research should systematically compare dogs across different populations, ages, and life experiences. Additionally, this study underscores the importance of refining methodological approaches to advance our understanding of reputation formation in animals.

## Electronic supplementary material

Below is the link to the electronic supplementary material.

Supplementary Video 1 (eavesdropping/control condition)

Supplementary Video 2 (direct experience condition)

Supplementary File 1 (full dataset)

Supplementary File 2 (tables and statistical analyses)

## Data Availability

All data supporting the findings of this study are available within the manuscript and its Supplementary Information.
