# Supplementary material for: Do dogs form reputations of humans? No effect of age after indirect and direct experience in a food-giving situation
Source: Anim Cogn. 2025 Jun 28;28(1):51. doi: 10.1007/s10071-025-01967-w (PMC12206192; doi:10.1007/s10071-025-01967-w)
Supplement: Supplementary file 4 — Supplementary File 2 (tables and statistical analyses) [file 10071_2025_1967_MOESM4_ESM.pdf]

Supplementary Information (SI) for:

Do dogs form reputations of humans? No effect of age after indirect and direct experience in a food-giving situation

Animal Cognition

Hoi-Lam Jim[1,2,3]*, Kadisha Belfiore[1], Eva B. Martinelli[1], Mayte Martínez[1,4], Friederike Range[1], Sarah Marshall-Pescini[1]*

[1]Domestication Lab, Konrad Lorenz Institute of Ethology, Department of Interdisciplinary Life Sciences, University of Veterinary Medicine Vienna, Vienna, Austria

[2]Institute for the Future of Human Society, Kyoto University, Kyoto, Japan

[3]Japan Society for the Promotion of Science, Tokyo, Japan

[4]Department of Psychology, University of Michigan, Ann Arbor, MI, USA

* Corresponding author

E-mail: hoi-lam.jim@outlook.com (HLJ)

E-mail: sarah.marshall@vetmeduni.ac.at (SMP)

Table S1 Individual characteristics of dogs that participated in the study

Name	Sex	Breed	Age (years)	Age group	Condition group	First condition	Previous experience in other experiments
Dunni	F	Miniature Pinscher	1	Young	Eavesdropping	Eavesdropping	Yes
Gandalf	M	Andalusian Hound	1			Eavesdropping	Yes
Crash	M	Australian Shepherd	2			Direct	Yes
Daytona	F	Australian Shepherd	2			Direct	No
Holly	F	Rottweiler	2			Direct	No
Ravenna	F	American Staffordshire Terrier	3			Eavesdropping	Yes
Ajani	F	Mixed breed	4	Adult		Eavesdropping	Yes
Cody	M	Siberian Husky	4			Eavesdropping	No

Chivas	M	Siberian Husky	5			Direct	Yes
Lilu	F	Mixed breed	5			Eavesdropping	No
Lenny	M	Canarian Warren Hound	6			Eavesdropping	Yes
Bailey	F	Pitbull Terrier	7			Direct	Yes
Capper	M	Andalusian Hound	7			Eavesdropping	Yes
Aaron	M	Labrador	8	Senior		Direct	Yes
Helena	F	Poodle	9			Eavesdropping	Yes
Sally	F	Mixed breed	9			Direct	No
Snoopy1	M	Shih Tzu	10			Eavesdropping	No
Amy	F	Border Collie	11			Direct	Yes
Monty	M	Border Collie	11			Direct	Yes

Snoopy2	M	Beagle	12			Direct	Yes
Jazzie	F	Labrador	1	Young	Control	Direct	No
Spaiky	M	Cane Corso	1			Direct	No
Franzi	F	Rottweiler	2			Direct	Yes
Timo	M	Mixed breed	2			Control	Yes
Zazu	F	Miniature Pinscher	2			Control	No
Asha	F	Rhodesian Ridgeback	4	Adult		Control	Yes
Sixtus	M	Petit Brabançon	4			Control	Yes
Django	M	American Staffordshire Terrier	5			Direct	Yes
Ellis	F	Portuguese Water Dog	5			Direct	No

Kiki	F	Mixed breed	6			Direct	Yes
Ozzy	M	Labrador	6			Control	No
Aidan	M	Irish Setter	6			Direct	Yes
Rusty	M	Mixed breed	7			Control	No
Lilly	F	Miniature Pinscher	8			Direct	Yes
Alois	M	Mixed breed	9			Direct	Yes
Fiona	F	Rhodesian Ridgeback	9			Control	Yes
Shari	F	Rottweiler	9	Senior		Direct	No
Jolie	F	Mixed breed	10			Control	Yes
Mozart	M	Labrador	11			Control	No
Ultimo	M	Border Collie	11			Control	Yes

Table S2 Results of the binomial GLM predicting partner choice in the baseline

Term	Estimate	SE	95% CI		Model stability		χ^2	*df*	*p*
			Lower	Upper	Min	Max			
Intercept	0.527	0.735	-0.908	2.053	0.107	0.815			[1]
Condition (Experimental)[2]	-1.331	0.693	2.767	-0.017	-1.464	-1.099	3.942	1	.047[3]
Age group (Adult)[2]	-0.360	0.851	-2.077	1.316	-0.661	-0.016	2.140	2	.343
Age group (Senior)	0.791	0.868	-0.895	2.566	0.452	1.094			

Estimate, standard errors (*SE*), 95% confidence intervals (CI), model stability (estimate ranges derived after excluding individuals one at a time) and results of significance tests

[1]The *p* value for the intercept is not shown due to its limited interpretability

[2]Reference level for factors are: Condition = Control, Age group = Young

[3]Although this *p* value is significant, the full-null comparison was not. Therefore, the estimate should not be interpreted as significant but as a likely false positive due to multiple testing within the model

Table S3 Results of the binomial GLMM predicting partner choice after the observation phase in the eavesdropping vs control condition

Term	Estimate	SE	95% CI		Model stability		χ^2	*df*	*p*
			Lower	Upper	Min	Max			
Intercept	-0.931	0.603	-0.183	0.211	-1.176	-0.651			[1]
Condition (Experimental)[2]	0.007	0.491	-0.960	0.975	-0.148	0.143	0.000	1	.989
Age group (Adult)[2]	0.094	0.618	-1.117	1.331	-0.155	0.247	1.208	2	.547
Age group (Senior)	0.627	0.635	-0.603	1.912	0.399	0.777			
Trial number (2)[2]	0.466	0.489	-0.488	1.442	0.355	0.609	0.913	1	.339
Condition order[3]	-0.340	0.254	-0.851	0.151	-0.410	-0.259	1.833	1	.176
~~Condition*Age group~~[4]							0.135	2	.935

Estimate, standard errors (*SE*), 95% confidence intervals (CI), model stability (estimate ranges derived after excluding individuals one at a time) and results of significance tests

[1]The *p* value for the intercept is not shown due to its limited interpretability

[2]Reference level for factors are: Condition = Control, Age group = Young, Trial number = 1

[3]z-transformed to a mean of 0 and a standard deviation of 1. Original mean (*SD*) = 1.500 (0.504)

[4]Removed from the model; the *p* value of the term at the time it was removed from the model is shown

Table S4 Results of the beta GLMM predicting time spent exhibiting affiliative behaviours towards the generous partner after the observation phase in the eavesdropping vs control condition

**Term**	**Estimate**	***SE***	**95% CI**		**Model stability**		χ^2	***df***	***p***
			**Lower**	**Upper**	**Min**	**Max**			
Intercept	0.387	0.359	-0.328	1.091	0.257	0.488			[1]
Condition (Eavesdropping)[2]	0.024	0.311	-0.587	0.635	-0.054	0.090	0.006	1	.937
Age group (Adult)[2]	-0.050	0.384	-0.806	0.706	-0.168	0.100	2.906	2	.234

Age group (Senior)	-0.603	0.397	-1.387	0.178	-0.751	-0.472			
Trial number (2)[2]	-0.127	0.310	-0.738	0.483	-0.230	-0.038	0.169	1	.681
Condition order[3]	0.039	0.158	-0.273	0.350	0.011	0.085	0.059	1	.807
~~*Condition*Age Group*~~[4]							1.110	2	.574

Estimate, standard errors (*SE*), 95% confidence intervals (CI), model stability (estimate ranges derived after excluding individuals one at a time) and results of significance tests

[1]The *p* value for the intercept is not shown due to its limited interpretability

[2]Reference level for factors are: Condition = Control, Age group = Young, Trial number = 1

[3]z-transformed to a mean of 0 and a standard deviation of 1. Original mean (*SD*) = 1.506 (0.503)

[4]Removed from the model; the *p* value of the term at the time it was removed from the model is shown

Table S5 Results of the binomial GLMM predicting partner choice after the experience phase in the direct experience condition

Term	Estimate	*SE*	95% CI		Model stability		χ^2	*df*	*p*
			**Lower**	**Upper**	**Min**	**Max**			
Intercept	-0.040	0.214	-0.477	0.393	-0.167	0.108			[1]
Age group (Adult)[2]	-0.040	0.285	-0.620	0.537	-0.196	0.096	0.565	2	.972
Age group (Senior)	-0.068	0.288	-0.655	0.512	-0.207	0.066			
Trial number[3]	-0.042	0.097	-0.232	0.148	-0.071	-0.009	0.185	1	.667
Condition order[3]	-0.039	0.115	-0.272	0.194	-0.083	0.004	0.117	1	.732
~~Trial number*Age group~~[4]							1.569	2	.456

Estimate, standard errors (*SE*), 95% confidence intervals (CI), model stability (estimate ranges derived after excluding individuals one at a time) and results of significance tests

[1]The *p* value for the intercept is not shown due to its limited interpretability

[2]Reference level for Age group = Young

[3]Continuous variables were z-transformed to a mean of 0 and a standard deviation of 1. Original mean (*SD*) are: Trial number = 6.477 (3.477), Condition order = 1.527 (0.500)

[4]Removed from the model; the *p* value of the term at the time it was removed from the model is shown

Table S6 Results of the beta GLMM predicting time spent exhibiting affiliative behaviours towards the generous partner after the experience phase in the direct experience condition

**Term**	**Estimate**	***SE***	**95% CI**		**Model stability**		χ^2	***df***	***p***
			**Lower**	**Upper**	**Min**	**Max**			
Intercept	0.276	0.136	0.007	0.556	0.225	0.312			[1]
Age group (Adult)[2]	0.146	0.179	-0.217	0.507	0.075	0.245	0.663	2	.718
Age group (Senior)	0.097	0.181	-0.270	0.461	0.015	0.182			
Trial number[3]	0.043	0.069	-0.094	0.179	0.025	0.059	0.376	1	.540

Condition order[3]	0.260	0.073	0.114	0.410	0.220	0.294	11.210	1	.001
~~Trial number*Age group~~[4]							0.241	2	.887

Estimate, standard errors (*SE*), 95% confidence intervals (CI), model stability (estimate ranges derived after excluding individuals one at a time) and results of significance tests

[1]The *p* value for the intercept is not shown due to its limited interpretability

[2]Reference level for Age group = Young

[3]Continuous variables were z-transformed to a mean of 0 and a standard deviation of 1. Original mean (*SD*) are: Trial number = 6.258 (3.458), Condition order = 1.495 (0.501)

[4]Removed from the model; the *p* value of the term at the time it was removed from the model is shown

Table S7 Results of the exact binomial tests for colour and side bias. Significant *p* values are in bold

Subject	Number of trials	Colour bias		Side bias	
		**Number of successes (black)**	***p***	**Number of successes (left)**	***p***
Aaron	12	6	1	1	**.006**
Aidan	15	8	1	7	1
Ajani	15	9	.607	6	.607
Alois	12	6	1	2	**.039**
Amy	12	11	**.006**	8	.388
Asha	15	8	1	8	1
Bailey	15	5	.302	5	.302
Capper	15	7	1	6	.607

Chivas	7	4	1	1	.125
Cody	14	4	.180	9	.424
Crash	14	8	.791	3	.057
Daytona	12	8	.388	3	.146
Django	15	7	1	6	.607
Dunni	15	3	**.035**	1	**.001**
Ellis	15	6	.607	4	.119
Fiona	14	8	.791	2	**.013**
Franzi	14	6	.791	4	.180
Gandalf	15	6	.607	8	1
Helena	15	7	1	0	**< .001**

Holly	15	9	.607	10	.302
Jazzie	15	10	.302	3	**.035**
Jolie	15	5	.302	12	**.035**
Kiki	10	8	.109	8	.109
Lenny	15	4	.119	6	.607
Lilly	15	7	1	4	.119
Lilu	15	5	.302	10	.302
Monty	15	6	.607	9	.607
Mozart	11	4	.549	6	1
Ozzy	15	8	1	14	**.001**
Ravenna	15	9	.607	4	.119

Rusty	15	7	1	2	**.007**
Sally	14	7	1	10	.180
Shari	12	6	1	6	1
Sixtus	15	9	.607	2	**.007**
Snoopy1	14	4	.180	3	.057
Snoopy2	13	3	.092	8	.581
Spaiky	14	9	.424	9	.424
Timo	13	3	.092	9	.267
Ultimo	15	9	.607	14	**.001**
Zazu	14	3	.057	5	.424

Supplementary Video 1 Example of the procedure for the baseline and observation phase in the control and eavesdropping conditions. The first clip shows the baseline (single trial). The second clip shows the subject (Django) observing the selfish partner (wearing white) and the generous partner (wearing black) interacting with an invisible dog in the control condition. The third clip shows another subject (Daytona) observing the two partners interacting with the dog demonstrator (Jasper) in the eavesdropping condition

Supplementary Video 2 Example of the procedure for the direct experience condition. The first clip shows the subject (Django) interacting with the generous partner (wearing black) and the selfish partner (wearing white) in the experience phase. The second clip shows two consecutive trials out of 12 in the test phase

Supplementary File 1 Full dataset used for statistical analyses

Supplementary File 2 Statistical analyses with the exclusion of dogs with a side bias

Fifteen dogs (5 young, 3 adult and 7 senior) that showed a side bias were excluded from the following analyses. This left a subset of 25 dogs (6 young, 12 adult and 7 senior). We repeated the analyses on choice and affiliative behaviours for both the eavesdropping vs control condition and the direct experience condition. However, since the sample size was reduced and the percentage of excluded dogs varied across age groups (i.e., a greater percentage of senior dogs (50%) showed a side bias compared to young (18%) and adult (40%) dogs), we treated age as a continuous predictor in these analyses.

We conducted four Generalized Linear Mixed-Effects Models (GLMMs):

Eavesdropping vs control condition:

1. **Binomial GLMM predicting partner choice after the observation phase:** Age (continuous variable) and condition (categorical variable), along with their two-way interaction, were included as predictors. Trial number (1 or 2) and condition order (i.e., whether dogs experienced the control/eavesdropping condition before or after the direct experience condition) were added as control variables. Trial number was treated as a factor with two levels, while condition order was treated as a continuous fixed effect. Subject ID was included as a random effect to account for repeated measures.
2. **Beta GLMM predicting proportion of time spent exhibiting affiliative behaviours towards the generous partner after the observation phase:** The predictors, their two-way interaction, control variables and random effect were identical to those used in the binomial GLMM described above.

Direct experience condition:

3. **Binomial GLMM predicting partner choice after the experience phase:** Age, trial number (1-12), and their two-way interaction were included as continuous predictors. Condition order was included as a continuous fixed-effect control variable. Subject ID was included as a random effect to account for repeated measures.
4. **Beta GLMM predicting proportion of time spent exhibiting affiliative behaviours towards the generous partner after the experience phase:** The predictors, their two-way interaction, control variable and random effect were identical to those used in the corresponding binomial GLMM described above.

We evaluated the quality of the models following the same steps as the main analyses. There were no issues with model stability, collinearity (maximum VIF = 1.370), overdispersion or Best Linear Unbiased Predictors (BLUPs).

1. Results of the binomial GLMM predicting partner choice after the observation phase in the *eavesdropping vs control condition*

After discarding the trials in which dogs did not choose either partner (5 trials), the data for this model comprised 45 trials from 24 dogs. The full model including age (linear and quadratic), condition, and its interaction was not better than the null model lacking condition (full-null model comparison, $\chi^2 = 3.667$, $df = 5$, $p = .598$; Table SI1). This indicates that neither age nor condition influenced the likelihood of choosing the generous partner.

Table SI1 Results of the binomial GLMM predicting partner choice after the observation phase in the eavesdropping vs control condition

**Term**	**Estimate**	***SE***	**95% CI**		**Model stability**		**LRT**	***df***	***p***
			**Lower**	**Upper**	**Min**	**Max**			
Intercept	-0.540	0.705	-2.026	0.806	-0.914	-0.228			[1]
Condition (Experimental)[2]	0.709	0.826	-0.863	2.443	0.371	1.060	0.764	1	.382
Age[3]	0.482	0.441	-0.325	1.467	0.309	0.863	1.317	1	.251
Age (quadratic)	-0.617	0.398	-1.487	0.107	-0.798	-0.448	2.745	1	.098
Trial number (2)[2]	-0.213	0.665	-1.547	1.094	-0.481	0.050	0.103	1	.748

Condition order[3]	-0.071	0.404	-0.887	0.731	-0.327	0.091	0.031	1	.860
*~~Age*Condition~~*[4]							0.000	1	.997
*~~Age(quadratic)*condition~~*[4]							0.001	1	.980

Estimate, standard errors (*SE*), 95% confidence intervals (CI), model stability (estimate ranges derived after excluding individuals one at a time) and results of significance tests

[1]The *p* value for the intercept is not shown due to its limited interpretability

[2]Reference level for factors are: Condition = Control, Trial number = 1

[3]Continuous variables were z-transformed to a mean of 0 and a standard deviation of 1. Original mean (*SD*) are: Age = 5.778 (2.190), Condition order = 1.578 (0.499)

[4]Removed from the model; the *p* value of the term at the time it was removed from the model is shown

2. **Results of the beta GLMM predicting proportion of time spent exhibiting affiliative behaviours towards the generous partner after the observation phase in the *eavesdropping vs control condition***

After discarding trials in which the subject did not spend any time exhibiting affiliative behaviours towards either partner (32 trials), the data for this model consisted of 43 trials from 24 dogs. The full model including age (linear and quadratic), condition, and its interaction was not better than the null model lacking condition (full-null model comparison, $\chi^2 = 2.930$, $df = 5$, $p = .711$; Table SI2). This indicates that neither age nor condition influenced the proportion of time that dogs spent exhibiting affiliative behaviours towards the generous partner.

Table SI2 Results of the beta GLMM predicting proportion of time spent exhibiting affiliative behaviours towards the generous partner after the observation phase in the eavesdropping vs control condition

**Term**	**Estimate**	***SE***	**95% CI**		**Model stability**		**LRT**	***df***	***p***
			**Lower**	**Lower**	**Min**	**Max**			
Intercept	0.154	0.410	-0.662	0.967	0.058	0.232			[1]
Condition (Experimental)[2]	-0.150	0.458	-1.061	0.754	-0.291	0.069	0.108	1	.743
Age[3]	-0.340	0.233	-0.806	0.120	-0.520	-0.228	2.117	1	.146
Age (quadratic)	0.034	0.210	-0.381	0.451	-0.075	0.108	0.026	1	.871

Trial number (2)[2]	0.312	0.396	-0.469	1.090	0.171	0.506	0.620	1	.431
Condition order[3]	0.058	0.231	-0.399	0.515	-0.016	0.213	0.063	1	.802
~~*Age*Condition*~~[4]							0.343	1	.558
~~*Age(quadratic)*condition*~~[4]							0.024	1	.878

Estimate, standard errors (*SE*), 95% confidence intervals (CI), model stability (estimate ranges derived after excluding individuals one at a time) and results of significance tests

[1]The *p* value for the intercept is not shown due to its limited interpretability

[2]Reference level for factors are: Condition = Control, Trial number = 1

[3]Continuous variables were z-transformed to a mean of 0 and a standard deviation of 1. Original mean (*SD*) are: Age = 5.744 (3.259), Condition order = 1.581 (0.499)

[4]Removed from the model; the *p* value of the term at the time it was removed from the model is shown

3. Results of the binomial GLMM predicting partner choice after the experience phase in the *direct experience condition*

After discarding the trials in which dogs did not choose either partner (29 trials), the data for this model comprised 271 trials from 25 dogs. The full model including age (linear and quadratic), trial number, and its interaction was not better than the null model lacking condition (full-null model comparison, $\chi^2 = 3.807$, $df = 5$, $p = .577$; Table SI3). This indicates that neither age nor experience with the partners influenced the likelihood of choosing the generous partner.

Table SI3 Results of the binomial GLMM predicting partner choice after the experience phase in the direct experience condition

Term	Estimate	*SE*	95% CI		Model stability		LRT	*df*	*p*
			Lower	Upper	Min	Max			
Intercept	-0.278	0.214	-0.729	0.156	-0.372	-0.178			[1]
Age[2]	0.024	0.156	-0.301	0.346	-0.071	0.139	0.024	1	.877
Age (quadratic)	0.110	0.156	-0.211	0.435	-0.065	0.195	0.497	1	.481
Trial number[2]	-0.102	0.125	-0.349	0.142	-0.140	-0.051	0.669	1	.413
Condition order[2]	0.021	0.151	-0.292	0.333	-0.036	0.090	0.020	1	.888

~~Age*Condition~~[3]							2.515	1	.113
~~Age(quadratic)*condition~~[3]							0.000	1	.984

Estimate, standard errors (*SE*), 95% confidence intervals (CI), model stability (estimate ranges derived after excluding individuals one at a time) and results of significance tests

[1]The *p* value for the intercept is not shown due to its limited interpretability

[2]Continuous variables were z-transformed to a mean of 0 and a standard deviation of 1. Original mean (*SD*) are: Age = 5.882 (3.291), Trial number = 6.454 (3.487), Condition order = 1.472 (0.500)

[3]Removed from the model; the *p* value of the term at the time it was removed from the model is shown

4. **Results of the beta GLMM predicting proportion of time spent exhibiting affiliative behaviours after the experience phase in the *direct experience condition***

After discarding trials in which the subject did not spend any time exhibiting affiliative behaviours towards either partner (69 trials), the data for this model consisted of 231 trials from 25 dogs. The full model including age (linear and quadratic), trial number, and its interaction was not better than the null model lacking condition (full-null model comparison, $\chi^2 = 4.057$, $df = 5$, $p = .541$; Table SI4). This indicates that neither age nor experience with the partners influenced the proportion of time that dogs spent exhibiting affiliative behaviours towards the generous partner.

Table SI4 Results of the beta GLMM predicting proportion of time spent exhibiting affiliative behaviours towards the generous partner after the experience phase in the direct experience condition

**Term**	**Estimate**	***SE***	**95% CI**		**Model stability**		**LRT**	***df***	***p***
			**Lower**	**Upper**	**Min**	**Max**			
Intercept	0.485	0.151	0.188	0.804	0.421	0.598			[1]
Age[2]	-0.019	0.109	-0.239	0.206	-0.087	0.034	0.031	1	.861
Age (quadratic)	-0.146	0.108	-0.370	0.070	-0.211	-0.052	1.816	1	.178
Trial number[2]	0.097	0.089	-0.077	0.272	0.071	0.119	1.192	1	.275

Condition order[2]	0.186	0.105	-0.025	0.403	0.128	0.244	3.032	1	.081
~~*Age*Condition*~~[3]							0.550	1	.458
~~*Age(quadratic)*condition*~~[3]							0.034	1	.854

Estimate, standard errors (*SE*), 95% confidence intervals (CI), model stability (estimate ranges derived after excluding individuals one at a time) and results of significance tests

[1]The *p* value for the intercept is not shown due to its limited interpretability

[2]Continuous variables were z-transformed to a mean of 0 and a standard deviation of 1. Original mean (*SD*) are: Age = 5.857 (3.289), Trial number = 6.264 (3.447), Condition order = 1.450 (0.499)

[3]Removed from the model; the *p* value of the term at the time it was removed from the model is shown
